# Change in Metabolic Profile after 1-Year Nutritional-Behavioral Intervention in Obese Children

**DOI:** 10.3390/nu7125520

**Published:** 2015-12-03

**Authors:** Elvira Verduci, Carlotta Lassandro, Roberta Giacchero, Vito Leonardo Miniello, Giuseppe Banderali, Giovanni Radaelli

**Affiliations:** 1 Department of Pediatrics, San Paolo Hospital, Department of Health Science, University of Milan, Milan 20142, Italy; carlotta.lassandro@unimi.it (C.L.); roberta.giacchero@ao-sanpaolo.it (R.G.); giuseppe.banderali@unimi.it (G.B.); giovanni.radaelli@unimi.it (G.R.); 2 Nutritional Sciences, University of Milano, Milan 20157, Italy; 3 Department of Pediatrics, Aldo Moro University of Bari, Giovanni XXIII Hospital, Bari 70126, Italy; vito.miniello@libero.it

**Keywords:** childhood obesity, nutritional-behavioral intervention, lipid profile, glucose metabolism, metabolic syndrome

## Abstract

Research findings are inconsistent about improvement of specific cardio-metabolic variables after lifestyle intervention in obese children. The aim of this trial was to evaluate the effect of a 1-year intervention, based on normocaloric diet and physical activity, on body mass index (BMI), blood lipid profile, glucose metabolism and metabolic syndrome. Eighty-five obese children aged ≥6 years were analyzed. The BMI *z*-score was calculated. Fasting blood samples were analyzed for lipids, insulin and glucose. The homeostatic model assessment of insulin resistance (HOMA-IR) was calculated and insulin resistance was defined as HOMA-IR >3.16. HOMA-β%, quantitative insulin sensitivity check index and triglyceride glucose index were calculated. The metabolic syndrome was defined in accordance with the International Diabetes Federation criteria. At the end of intervention children showed a reduction (mean (95% CI)) in BMI *z*-score (−0.58 (−0.66; −0.50)), triglycerides (−0.35 (−0.45; −0.25) mmol/L) and triglyceride glucose index (−0.29 (−0.37; −0.21)), and an increase in HDL cholesterol (0.06 (0.01; 0.11) mmol/L). Prevalence of insulin resistance declined from 51.8% to 36.5% and prevalence of metabolic syndrome from 17.1% to 4.9%. Nutritional-behavioral interventions can improve the blood lipid profile and insulin sensitivity in obese children, and possibly provide benefits in terms of metabolic syndrome.

## 1. Introduction

Childhood obesity has become a worldwide concern, affecting children of high-income countries as well as middle-income and low-income countries [1]. Although recent studies suggested that progresses have been made in the control of the obesity epidemic [2,3] the prevalence of childhood obesity remains high [1]. Obese children are exposed to detrimental short and long-term effects on health, thus showing components of metabolic syndrome [4], such as dyslipidemia [5], hypertension [6], insulin resistance and disturbed glucose metabolism [7]. In most cases, obesity is consequence of a chronic imbalance between energy intake and energy expenditure, involving environmental and lifestyle factors, e.g., easy access to energy-dense foods, increased portion sizes, reduced physical activity and increased time spent in sedentary activities [8]. Chronic exposure over time to these factors may potentiate weight gain over many years [8].

Guidelines for treatment of childhood obesity recommend intensive lifestyle interventions, involving diet, behavioral and physical activity for the child and the entire family, in an age-appropriate manner [9]. A Cochrane systematic review stated that in children, family-based lifestyle interventions aimed at changing dietary, behavioral and physical activity patterns can lead to a reduction in overweight, compared to standard care or self-help [10]. A meta-analysis of randomized trials conducted on overweight/obese children showed a small to moderate effect from combined lifestyle interventions on body mass index (BMI) [11]. The largest effects were observed when lifestyle modifications were implemented with parental involvement [11]. In a recent meta-analysis, Ho *et al.* [12] evaluated randomized controlled trials with a follow-up period of at least 2 months from baseline and highlighted that lifestyle interventions, incorporating diet and physical exercise and/or behavioral treatment, can lead to improvement in weight and cardio-metabolic outcomes, compared to no treatment/wait-list control, usual care, or written education materials. These authors also reported that although both diet-only and combined interventions (diet plus exercise) may result in weight loss and metabolic improvement in the overweight/obese pediatric population, combined interventions can determine larger improvement in levels of high-density lipoprotein (HDL) cholesterol, fasting glucose and insulin over 6 months [13]. Other authors suggested that improved weight status may be achieved by a reduced-energy diet, but the need for an adequate content of macronutrients has to be considered when aiming at specific cardio-metabolic risk factors [14]. Studies evaluated the effect of a lifestyle intervention, in overweight/obese children, characterized by nutritional counseling and education, within an intervention period ranging from 20 weeks to 12 months [15,16,17,18,19]. While a decrease in BMI *z*-score has been observed [15,16,17,18,19], research findings are inconsistent about improvement of specific cardio-metabolic variables.

The primary aim of this study was to evaluate whether a 1-year intervention based on normocaloric diet and physical activity may impact the BMI status, blood lipid profile and glucose metabolism indicators in obese children. Additionally, metabolic syndrome was assessed.

## 2. Experimental Section

A cohort of 90 obese children (44 boys and 46 girls) was consecutively recruited among those admitted with diagnosis of obesity by primary care pediatricians to the Department of Pediatrics, San Paolo Hospital, Milan, Italy, between 1 January 2012 and 31 December 2014, according to the following eligibility criteria: age ≥6 years, weight at birth ≥2500 g and <4000 g, gestational age 37–42 weeks, single birth, children having white parents and residing in Milan or neighborhood (<30 km). Children having syndromic, organic and hormonal conditions besides obesity were excluded.

A child was defined obese in accordance with the International Obesity Task Force, *i.e.*, if her/his BMI was above the age- and sex-adjusted BMI Cole’s curve passing through the cut-off of 30 kg/m^2^ at age 18 years [20]. The parents of eligible children or their legal guardian received detailed explanation about the aim of the study, and signed a consent form. The Hospital Ethics Committee approved the study protocol and gave ethical clearance.

### 2.1. Anthropometry and Blood Pressure

A medical history was collected at recruitment from parents by a standardized questionnaire during a personal interview conducted by the same pediatrician that saw children for a general examination and evaluated the Tanner stage of puberty [21]. The pediatrician also took anthropometric measurements and blood pressure of children both at recruitment and at the end of intervention, assisted by an experienced operator. Body weight and height were measured using a mechanical column scale (seca 711; seca GmbH & KG, Hamburg, Germany) with integrated measuring rod (seca 220; seca GmbH & KG). BMI was calculated from the ratio of weight to height squared (kg/m^2^). BMI *z*-scores were calculated and adjusted for age and sex by using Cole’s LMS method [22] and Italian reference data [23]. Waist circumference (WC) was measured using the measuring tape seca 203 (seca GmbH & KG) to the nearest 0.1 cm at the mid-point between the iliac crest and the lower edge of the ribs at the end of a normal expiration. Triceps skinfold thickness was measured on the left side of the body, using the Harpenden Skinfold Caliper (Chasmors Ltd, London, UK) halfway between the acromion process and the olecranon process [24]. Blood pressure was measured according to recommendations of the National High Blood Pressure Education Program Working Group [25].

### 2.2. Biochemistry

Biochemical measurements were performed within 3 ± 1 day (baseline) of recruitment and one year (±5 day) after starting intervention (end of intervention). Fasting blood samples were taken at 8 h ± 30 min a.m. and immediately analyzed at the hospital laboratory of biochemistry for total cholesterol, HDL cholesterol, low-density-lipoprotein (LDL) cholesterol, triglycerides, apolipoprotein A1, apolipoprotein B, insulin and glucose on the cobas^®^ 6000 analyzer series, c501 and e601 modules (Roche Diagnostics GmbH, Hoffmann-La Roche ltd, Mannheim, Germany), which has been recognized as providing robust chemistry and immunochemistry [26]. The homeostatic model assessment of insulin resistance (HOMA-IR) was calculated as the product of fasting glucose (mmol/L) and fasting insulin (µU/mL) divided by 22.5 [27], and insulin resistance was defined as HOMA-IR >3.16 [28]. The quantitative insulin sensitivity check (QUICK) index was calculated as 1/(log_10_ fasting plasma insulin in µU/mL + log_10_ glucose in mg/dL) [29]. Pancreatic β-cell function was evaluated by HOMA-β% as (20 × fasting insulin in µU/mL)/(fasting glucose in mmol/L − 3.5) [27]. The triglyceride glucose index (TyG index) was calculated as ln [fasting triglycerides (mg/dL) × fasting glucose (mg/dL)/2] [30,31].

### 2.3. Dietary Habits

Dietary habits of children were assessed at baseline and at the end of intervention by a food frequency questionnaire (FFQ) originally developed at our Department in 1990’s on the original Block FFQ [32] and then revised and updated in 2008 on the basis of the full-length Block 2005 FFQ © (NutritionQuest, Berkeley, CA, USA) and the 2007 national food composition tables [33], to appropriately adjust for cultural food/beverage items of the Italian pediatric population. Parents completed the FFQ about their children’s habits during an interview of approximately 50 min, conducted at hospital by the same experienced dietitian unaware of the obesity status of children. Each meal was analyzed to find out which food was eaten and how often. Usual portion sizes were estimated using household measures and the weight (e.g., pasta) or unit (e.g., fruit juice) of the purchase. A 24-h recall was additionally recorded at the end of the interview to standardize the usual serving size. Quantification and analysis of the energy intake and nutrient composition were performed with an *ad hoc* PC software program developed by a consultant.

### 2.4. Metabolic Syndrome

Metabolic syndrome was defined in accordance with the International Diabetes Federation (IDF) criteria for children and adolescents [34,35]. As IDF suggests that below the age of 10 years metabolic syndrome cannot be diagnosed [34,35], in this study it was evaluated only in children of 10 years or older.

### 2.5. Intervention

The intervention was based on promotion of a normocaloric diet, balanced for the macronutrient distribution, in accordance with the national guidelines for treatment of childhood obesity [36]. Specifically, it was recommended that children follow, for a 1-year period, a normocaloric diet (daily caloric intake by age and sex [37]) consisting of protein (12%–15%), carbohydrates (55%–60%), fat (25%–30%; <10% saturated fatty acids, polyunsaturated up to 10%, monounsaturated up to 15%) and fiber (range: age (year) plus 5 g–age (year) plus 10 g) [36,37]. Additionally, it was recommended that children engage in at least 60 min of moderate- to vigorous-intensity physical activity (MVPA) daily [38], based on walking, and tailored to individual preferences. MVPA was estimated using 3-day physical activity recall (3DPAR). During a first round 1-h educational session, held at hospital on the day of recruitment, a pediatrician and an experienced dietitian provided illustration and instructed parents and children about the intervention to be performed and actions to maintain through a 1-year period. Education was based and focused on regulation of energy expenditure, body composition, physical activity, consequences of sedentary lifestyle, principles of nutrition, food sources, glycemic index and glucose metabolism, to continuously enhance and maintain parental and self-efficacy for dietary change. This education managing also took into account a range of behavior change techniques from the revised CALO-RE taxonomy (items 1, 2, 5, 6, 8, 16, 21, and 26) [39]. In particular, written guidelines were given to the parents, including general nutritional advice, food choice lists, selected week menu, and recommended average servings for principal food categories, according to age and sex. General nutritional advice included increasing fruit and vegetable intake, increasing legume and fish intake while decreasing meat consumption, using more whole grain food, avoiding sugary beverages and limiting sweets. Educational and incentive documentation (friendly, illustrated brochures) about potential benefits of a routinely normocaloric diet and physical activity for the child and family were also given to parents, together with a diary for recording the physical activity of their child, in terms of type, frequency, duration and intensity. A second explanatory session tailored for parents requests was held at the hospital on the day of blood sampling (*i.e.*, within 3 ± 1 day of recruitment) to resolve any doubts parents had about intervention, providing them with an instructive point-by-point reply. Lastly, the study design scheduled a dietitian to contact the parents by phone on a midweek day at 3-month intervals to fill out a 24-h recall and ask about the physical activity of the child as recorded in the diary. Parents were also invited to actively contact a pediatrician by phone (8–20 h) at any time of the intervention, when necessary.

### 2.6. Outcomes

The primary outcome measures were the change in BMI *z*-score and HDL cholesterol at the end of intervention. Secondary measures were the change in the other blood lipid variables and insulin resistance, and in the prevalence of metabolic syndrome.

### 2.7. Sample Size

The sample size was calculated iteratively to detect a mean longitudinal variation of 5% or more of HDL cholesterol, based on the baseline mean and standard deviation estimated in children already recruited. Assuming a type I error level of 0.05 with a power of 0.80, and allowing for a drop-out of 5% at least 87 children needed to be recruited.

### 2.8. Statistical Analysis

Descriptive data are reported as mean and standard deviation (SD) or 95% confidence interval (CI), or number of observations (percentage). Normality of the distribution of continuous variables was assessed by the Kolmogorov–Smirnov test. Means were adjusted for age, sex and baseline BMI *z*-score, as appropriate. Statistical significance of longitudinal variations was tested by the Student’s *t* test for paired data or the Wilcoxon test, and also adjusted by ANOVA for repeated measures. At this analysis non-normally distributed continuous variables entered the model after logarithmic transformation. All values of *p* < 0.05 were considered to indicate statistical significance (two-tailed test). The statistical package for social sciences (SPSS) package version 20.0 (SPSS Inc., Chicago, IL, USA) for Windows (Microsoft, Redmond, WA, USA) was used, for the statistical analysis.

## 3. Results

Eighty-five children (94.4%), 42 boys and 43 girls, completed the intervention. At recruitment, mean (SD) age and duration of obesity were 9.7 (2.6) years (range 6–15) and 4.0 (2.1) years, respectively. At the end of intervention there was a reduction of daily energy intake and macronutrient redistribution towards the recommended range (Table 1). Mean (SD) MVPA was 45.4 (33.2) min/day at baseline and 54.7 (35.0) min/day at the end of intervention (*p* = 0.089). No change was observed for systolic (113.33 (11.2) *vs.* 112.8 (9.5) mmHg; *p* = 0.524) and diastolic (68.47 (9.25) *vs.* 67.83 (7.52) mmHg; *p* = 0.321) blood pressure.

**Table 1:** Daily dietary intake of energy, macronutrients and fiber, and overall glycemic index and glycemic load at baseline and at the end of intervention. Values are mean (SD) ^†^.

Variable	Baseline (*n* = 85)	End of Intervention (*n* = 85)	*p*-Value	Recommended Intake [36,37]
Energy				
kcal/day	2460.46 (795.84)	1855.62 (614.31)	<0.001 *	1380–3330 kcal/day depending on age and sex (6–15 year)
kcal/kg/day	45.43 (18.86)	34.39 (16.94)	0.001 *	
Protein				
g/day	96.32 (29.50)	71.12 (23.74)	<0.001 *	
% Energy	15.82 (3.02)	15.54 (3.16)	0.006 *	12%–15% Energy
Carbohydrates				
g/day	332.69 (113.51)	268.04 (106.59)	<0.001 *	
% Energy	54.20 (7.19)	58.07 (9.62)	0.001 *	55%–60% Energy
Fats				
g/day	84.54 (37.94)	57.27 (21.04)	<0.001 *	
% Energy	31.44 (5.48)	27.97 (4.16)	<0.001 *	25%–30% Energy
Saturated				
g/day	31.64 (14.40)	19.48 (8.36)	<0.001 *	
% Energy	11.69 (3.07)	9.44 (3.15)	<0.001 *	<10% Energy
Monounsaturated				
g/day	34.53 (15.49)	22.31 (9.28)	<0.001 *	
% Energy	12.64 (2.87)	10.80 (3.52)	0.022 *	≤15% Energy
Polyunsaturated				
g/day	14.92 (7.20)	9.83 (3.93)	<0.001 *	
% Energy	5.40 (1.57)	4.72 (1.74)	0.016 *	≤10% Energy
Fiber g/day	11.30 (5.15)	17.11 (8.02)	<0.001 *	age (year) plus 5 g–age (year) plus 10 g
Overall Glycemic Index	43.58 (22.42)	41.57 (22.32)	0.146	
Glycemic Load	418.73 (565.56)	307.56 (377.64)	0.124	

^†^ Mean and *p*-value adjusted for age and sex; * Statistically significant.

**Figure 1:**
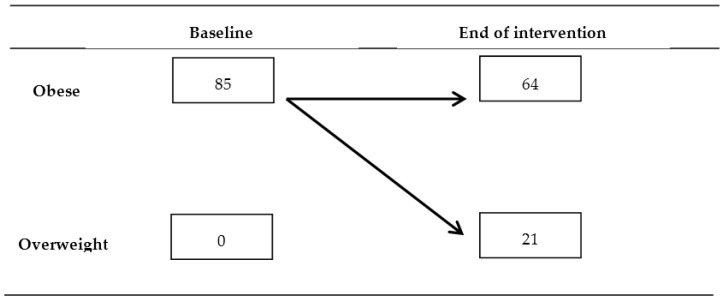
Within-subject longitudinal variation of obesity status through the intervention period (figures are number of children). Significance of longitudinal variation was *p* < 0.0001 (Wilcoxon test).

At the end of intervention children showed lower BMI *z*-score than at recruitment (2.96 (0.96) *vs.* 3.54 (1.04); *p* < 0.0001) and lower triceps skinfold thickness (24.05 (5.74) *vs.* 27.18 (5.42) mm; *p* < 0.038), while no difference was found for waist circumference (81.76 (9.88) *vs.* 83.69 (10.68) cm; *p* = 0.150). The within-subject longitudinal variation of obesity status was significant (Figure 1).

HDL cholesterol increased through the intervention period (mean variation, Δ, 0.06; 95% CI, (0.01; 0.11) mmol/L) while there was a reduction in triglycerides (−0.35; (−0.45; −0.25) mmol/L) and triglycerides/HDL cholesterol ratio (Δ = −0.36; (−0.46; −0.26)) (Table 2). Reduction in triglyceride glucose index (−0.29; (−0.37; −0.21)) and prevalence of insulin resistance were observed (Table 3).

**Table 2:** Blood lipid profile at baseline and at the end of intervention. Values are mean (SD) ^†^.

Variable	Baseline (*n* = 85)	End of Intervention (*n* = 85)	*p-*Value
Total cholesterol (mmol/L)	4.40 (0.62)	4.21 (0.70)	0.072
LDL cholesterol (mmol/L)	2.58 (0.58)	2.50 (0.65)	0.116
HDL cholesterol (mmol/L)	1.26 (0.21)	1.32 (0.26)	0.034 *
Triglycerides (mmol/L)	1.29 (0.66)	0.94 (0.40)	0.024 *
Apo A1 (g/L)	1.33 (0.23)	1.34 (0.21)	0.772
Apo B (g/L)	0.76 (0.19)	0.70 (0.17)	0.164
ApoB/ApoA	0.58 (0.18)	0.54 (0.16)	0.546
Triglycerides/HDL cholesterol	1.13 (0.77)	0.77 (0.45)	0.018 *
LDL/HDL cholesterol	2.14 (0.80)	1.99 (0.76)	0.181
Total cholesterol/HDL cholesterol	3.62 (0.95)	3.34 (0.95)	0.078

LDL, low-density-lipoprotein; HDL, high-density-lipoprotein; Apo A1, apolipoprotein A1; Apo B, apolipoprotein B. SI conversion factors: to convert cholesterol, divide values by 0.0259; to convert triglycerides, divide values by 0.0113; to convert Apo A1 and Apo B divide values by 0.01. ^†^ Mean and *p*-value adjusted for age, sex, baseline BMI *z*-score. * Statistically significant.

**Table 3:** Glucose metabolism variables at baseline and at the end of intervention. Values are mean (SD) ^†^ or number of children (percentage).

Variable	Baseline (*n* = 85)	End of Intervention (*n* = 85)	*p-*Value
Glucose (mmol/L)	4.78 (0.33)	4.74 (0.29)	0.341
Insulin (pmol/L)	133.28 (102.22)	98.64 (59.51)	0.399
HOMA-IR	4.10 (3.30)	3.01 (1.92)	0.281
HOMA-β%	317.10 (221.23)	237.61 (123.26)	0.368
QUICK index	0.32 (0.03)	0.33 (0.03)	0.250
TyG index	8.38 (0.51)	8.09 (0.43)	0.030 *
Insulin resistance (yes)	44 (51.8)	31 (36.5)	0.008 *^,††^

HOMA-IR, homeostasis model assessment of insulin resistance; QUICK, quantitative insulin sensitivity check; TyG, triglyceride glucose. SI conversion factors: to convert glucose, divide values by 0.0555; to convert insulin, divide values by 6.945. Insulin resistance (yes): HOMA-IR >3.16. ^†^ Mean and *p*-value adjusted for age, sex, baseline BMI *z*-score. ^††^ Wilcoxon test. * Statistically significant.

At the end of intervention, prevalence of metabolic syndrome was reduced by 71.4% (Table 4). No component worsened for any child. The only child who had waist circumference decreased below the 90th percentile was not syndromic at baseline. Fourteen children recovered one of the other metabolic components and two children recovered 2 components.

At 3, 6, 9 and 12 months of intervention, compliance with diet and MVPA, evaluated by the 24-h recall (plus FFQ at 12 months) and diary of physical activity, was 84%, 86%, 88% and 92% (diet) and 83%, 86%, 85% and 87% (MVPA), respectively.

**Table 4:** Distribution of children according to International Diabetes Federation criteria for metabolic syndrome ^†^ [34,35] at baseline and at the end of intervention. Values are number of children (percentage).

	Baseline (*n* = 41)	End of Intervention (*n* = 41)	*p-*Value ^††^
Metabolic syndrome	7 (17.1)	2 (4.9)	0.025 *
Component			
Waist circumference ≥90th percentile	40 (97.6)	39 (95.1)	0.317
Triglycerides ≥1.7 mmol/L	10 (24.4)	3 (7.3)	0.008 *
HDL cholesterol <1.03 mmol/L	12 (29.3)	7 (17.1)	0.025 *
Blood pressure: Systolic ≥130/Diastolic ≥85 mmHg	9 (22)	3 (7.3)	0.034 *
Glucose ≥5.6 mmol/L	0 (0)	0 (0)	1.000

HDL, high-density-lipoprotein. SI conversion factors: to convert cholesterol, divide values by 0.0259; to convert triglycerides, divide values by 0.0113. ^†^ Evaluated in children aged ≥10 years (20 boys, 21 girls). Diagnosis of metabolic syndrome requires waist circumference ≥90th percentile and two or more of the other components. ^††^ Wilcoxon test. * Statistically significant.

## 4. Discussion

This longitudinal study evaluated whether a 1-year nutritional-behavioral intervention, based on normocaloric balanced diet and physical activity, may impact the BMI status and metabolic profile of obese children aged ≥6 years. The participation rate was high, ranging from 100% at baseline to 94.4% at the end of intervention. Strict international definitions and accurate anthropometric and biochemistry measurement procedures were used. Compliance with treatment, as based on national recommended dietary energy and macronutrient intakes [36,37], was acceptable, with more than 90% of children who recovered at the end of intervention towards the recommended range. However, owing to the study design, which did not include a control group of obese children on a free diet, and based on the dietary assessment on the Food Frequency Questionnaire, caution should be exercised in drawing definitive conclusions. Indeed, it should be pointed out that while the absence of a control group on a free diet is a limitation, the recruitment of such a group was discouraged by the Hospital Ethics Committee due to the opinion that all obese children and their families should have the same opportunity to be instructed about dietary recommendations, while also taking into account the current international guidelines [9]. The use of the FFQ and 24-h recall, instead of a three- or seven-day food diary, was chosen as it provides for immediate collection of data and because the dietary assessment was primarily planned to estimate compliance with intervention and not its effect size. Another limitation is that the study did not fully meet the revised CALO-RE taxonomy [39]. Indeed, while the revised CALO-RE taxonomy was not available before starting this study, only a subset of items was extracted by a consultant psychologist from the original 26-item taxonomy [40] due to financial constraints. Regardless, it should be noted that most of the selected items have been recently recognized as providing effective behavior change techniques for childhood obesity [41].

At the end of intervention, children showed a decrease in mean BMI *z*-score of 16%, and 25% of them recovered from obesity to overweight. Other studies [15,16,17,18,19,42,43] conducted on overweight/obese children found, at the end of nutritional/lifestyle interventions, a decrease in mean BMI *z*-score, ranging from about 5% [18,19] to 20% [15]. In this study no overall significant change was detected for waist circumference while a decrease in triceps skinfold thickness was observed. It should be pointed out that skinfold thickness is *de facto* a measure of subcutaneous fat unable to quantify visceral adiposity, while waist circumference is a useful indicator for identifying children at increased risk of cardiovascular disease and metabolic syndrome [44]. The authors observed a waist circumference decrease in obese/overweight children after lifestyle interventions based on hypocaloric diet [43] or not [15,16,19] while no change of waist circumference was detected in obese/overweight children aged 7–9 years after a 1-year lifestyle intervention based on a recommended dietary allowance of about 1800 kcal or nutrition education program [17]. A study conducted on 484 children who underwent a 1-year intervention based on physical activity, nutrition education, and behavior therapy found decreased blood pressure [16]. In this study no significant change was observed in mean blood pressure, as was also found in a trial that evaluated the effects of a 20-week exercise and diet guidance intervention on 19 overweight school-aged children [15].

HDL cholesterol and triglycerides have a key role in cardiovascular disease. HDL cholesterol protects against vascular disease by removing the “bad” cholesterol from the walls of arteries while high triglycerides increase the risk of atherosclerotic cardiovascular disease [45]. In our study, increased HDL cholesterol and decreased triglycerides levels were found. While these findings agree with other studies [16,17,43], it should be noted that a recent systematic review and meta-analysis examining the impact of lifestyle interventions, including dietary caloric restrictions and/or nutrition education, on cardio-metabolic risk factors in overweight/obese children reported that fewer than half of evaluated studies demonstrated significant improvements in HDL cholesterol or triglycerides levels [12]. The same authors [13] suggested that diet plus exercise interventions may produce greater improvement in HDL cholesterol than diet-only interventions. Concerning the other lipid variables, no improvement in total cholesterol, LDL cholesterol, Apolipoprotein A1 and Apolipoprotein B was observed while other authors reported a significant change of at least one of these lipid variables [16,17,18,43].

Lifestyle interventions based on a hypocaloric diet [43] or not [16,18] reported decreased insulin and/or HOMA-IR after one year of intervention. In this study, the change in insulin, HOMA-IR, HOMA-β% and QUICK index was not statistically significant. However it should be noted that the prevalence of insulin resistance decreased by 30%, from a baseline value of 51.8%, which is comparable with estimates reported in the literature, ranging from 32% [46] to 52% [47]. Triglyceride glucose index is an emergent useful indicator, affording an easily and widely available simple laboratory method as a surrogate to estimate the insulin sensitivity [30,31]. Only one study examined its usefulness in pediatric age, suggesting that it could be used in the metabolic evaluation of obese adolescents [31]. To our knowledge this study is the first intervention study that evaluated triglyceride glucose index in a pediatric population, and found that it decreased at the end of the intervention. This result also suggests that assessment of triglyceride glucose index might be included in future research investigating on glucose-metabolism alterations in obese children.

Metabolic syndrome was firstly defined in adult population as “a link between insulin resistance, hypertension, dyslipidemia, impaired glucose tolerance and other metabolic abnormalities associated with an increased risk of athero-sclerotic cardiovascular diseases” [48] and it has also been successively defined in the pediatric population. Recently, the International Diabetes Federation has suggested a unified definition that can be profitably used in children [35]. In this study, the prevalence of metabolic syndrome, defined according to IDF criteria [34,35], was 17% and 5% at, respectively, the baseline and the end of intervention. Reinehr *et al.* [49], when using the IDF definition, found in obese children a comparable decline of prevalence, going from 19% to 9% after 1-year lifestyle intervention. Other authors found a variation of prevalence from 17% to 10%, after 1-year lifestyle intervention characterized by a diet with a caloric intake 250–500 kcal less per day than the daily requirement, but variation was not statistically significant [43].

Concerning specific components of metabolic syndrome, the percentage of children with blood level of triglycerides ≥1.7 mmol/L, lower levels of HDL cholesterol and high systolic or diastolic blood pressure significantly declined by 70%, 42% and 67%, respectively. In the study conducted by Reinehr *et al.* [49] variation of IDF components was significant for blood pressure only, with a reduction of about 50%. Direct comparison with findings reported in other studies is not here possible due to the different adopted definition of the metabolic syndrome (e.g., [17]).

On the whole, within the limitations of this study, one may conclude that in obese children, interventions based on normocaloric diet and physical activity could result in a decrease of BMI *z*-score and also benefit blood lipid profile and insulin sensitivity. Additionally, it might play a positive role in terms of metabolic syndrome. Large longitudinal trials with adequate power, and hopefully meeting the revised CALO-RE taxonomy, are desirable to better evaluate the clinical relevance and long-term effectiveness of nutritional-behavioral interventions based on normocaloric diet and physical activity.
